# Development of Liquid Chromatography on Monolithic Supports—From First Concepts to Real Analytical and Preparative Techniques

**DOI:** 10.3390/ijms26104695

**Published:** 2025-05-14

**Authors:** Tomislav Friganović, Djuro Josić

**Affiliations:** 1 Genos Glycoscience Research Laboratory, 10000 Zagreb, Croatia; 2 Laboratory of Clinical Chemistry, Faculty of Medicine, Juraj Dobrila University, 52100 Pula, Croatia

**Keywords:** monolithic chromatography, convective interaction media (CIM), bioprocessing scale-up, immobilized-enzyme reactors, biomolecule purification, protein purification, plasmid DNA purification, virus purification, extracellular vesicles, historical development of monoliths

## Abstract

In this review, we trace the evolution of liquid chromatography from the pioneering work of Tennikova and Svec to the current monolithic polymethacrylate supports for performing liquid chromatography with biological macromolecules and nanoparticles, which offer rapid, high-throughput separations. By using interconnected channels with a tailored channel diameter, monoliths minimize the diffusion limitations typical of particle-based systems. Radial flow designs and optimized channel architectures enable the direct loading of complex biological fluids, reducing the need for sample preparation and optimizing the purification of large biomolecules and nanoparticles such as proteins, nucleic acids, extracellular vesicles, and viruses. Recent work has integrated monoliths into immunoaffinity and enzyme reactor platforms, streamlining analytical workflows and preparative applications in vaccine production and gene therapy. The ongoing advances in monolithic materials, channel geometry, and continuous processing hold promise for even greater efficiency and scalability in future applications.

## 1. Introduction

Liquid chromatography is a separation technique in which the components of a mixture partition differently between the stationary phase and the moving mobile phase, causing them to migrate at distinct velocities. This differential migration translates molecular interactions into spatial resolution, allowing both the identification and purification of target species.

Historically, chromatography dates back to the early 1900s, when Mikhail Tswett introduced column adsorption methods for separating plant pigments and coined the name “chromatography” [1]. In the 1930s, Kuhn and Lederer demonstrated utility of this method for carotenoid separation [2,3]. The next step happened in 1943 when Arne Tiselius discovered displacement chromatography [4,5]. Initially, this approach was applied for the separation of amino acids [5,6], and Horváth’s group refined it further in the late 1970s [7,8,9].

In the late 1950s, Porath and Flodin introduced size exclusion chromatography (SEC) [10]. This method enables the gentle separation of large molecules based on their hydrodynamic volumes, generally preserving the biological activity of the biomolecules [11]. Practical and industrial applications became intensive in the 1970s, with process chromatography being applied for the industrial separation of low- and high-molecular-weight milk components [12]. These developments paved the way for the modern chromatography of biopolymers, then nanoparticles, and then to the isolation of increasingly larger molecules and macromolecular structures, such as extracellular vesicles, cell organelles, and viruses [13,14,15,16]. The benefits of newly optimized monolithic supports have become more and more notable for the latter kind of separation [17].

The 1951 work of Campbell et al. established the foundation for immunoaffinity chromatography (IAC) by immobilizing serum albumin on *p*-aminobenzylcellulose to purify antibodies [18]. Historically, it was the first chromatographic support that was used for the affinity chromatography of biomolecules. According to Campbell et al., “Methods are described for the preparation and use of an insoluble antigenic adsorbent obtained by coupling protein to diazotized *p*-aminobenzylcellulose”. The data that were presented indicated the practical usefulness of such a method for isolation and purification, suggesting that such chromatographic techniques can be applied for the separation of antibodies that are specific for target antigen(s). However, the human serum albumin that was used as an immobilized ligand did not have the necessary binding specificity. This problem was solved by use of protein A as well as similar bacterial and recombinant proteins (cf. below) with high specific affinity to immunoglobulins [19].

The subsequent work of Martin and Synge [19,20,21] on improving separation efficiency led to additional advances in liquid chromatography methods. By the 1960s, this progress had led to high-performance liquid chromatography (HPLC), which was aided by the development of microparticulate and other more sophisticated high-performance stationary phases like non-porous microparticles [22] as well as significantly improved instrumentation [23].

In the 1970s and 1980s, IAC became a widely used method in bioanalysis and biotechnology, with applications in purification, immunodepletion, direct sample analysis, and efficient chromatographic immunoassays [24]. An important breakthrough was the introduction of protein A and protein G affinity chromatography, which allowed for the efficient purification of antibody-based targets by binding to the fragment crystallizable region of antibodies [24,25,26,27]. Protein A and protein G became widely used for selective protein purification, as well as for the isolation and enrichment of other biomolecules and nanoparticles [24,28], playing an important role in clinical diagnostics and protein- and nucleic-acid-based therapeutics [29,30,31].

A noteworthy breakthrough was the introduction of synthetic resins, which enabled the more efficient ion exchange chromatography for amino acids and proteins [32]. However, the chromatographic separation of proteins and other biologically active biopolymers like nucleic acids still faced challenges caused by small pore diameters and undesired surface interactions, and denaturation on support surfaces. One of the main problems was the recovery of physiologically active proteins, especially hydrophobic ones, and other biomolecules while preserving their initial function and activity. Regnier drew attention to these issues and stressed the importance of pore design and surface modifications [33]. Enlarged pores and rigid supports enhanced mass transfer and improved separation kinetics, reducing the irreversible adsorption and minimizing the conformational changes that could lead to protein denaturation [22]. As HPLC gained popularity, reversed-phase chromatography (RPC) was widely adopted for peptide and protein separations due to its high resolution and selectivity [34,35]. RPC became also a standard technique for protein purification and analysis [34,36]. However, the organic solvents involved in the separation process may compromise protein integrity and consequently biological activity and immunogenicity [37]. To overcome these barriers, hydrophobic interaction chromatography (HIC) was proposed. HIC retains proteins in high-salt environments and elutes them with decreasing ionic strength. However, partial denaturation still occurs, especially with sensitive proteins [38]. Over time, the development of stationary phases mitigated these drawbacks, and both RPC and HIC became valuable tools in separation of biomolecules [38,39].

The development of biotechnology, especially in the field of the production of recombinant therapeutics and in the fractionation of other biological fluids, also boosted the development of supplemental methods in the separation sciences, in the fields of both instruments and chromatographic supports. However, the separation of complex biological fluids like cell culture supernatants, blood plasma, or other frequently complex and highly viscous and sometimes even non-Newtonian fluids that contain complex and partially hydrophobic molecules was still a challenge. The use of newly developed SEC columns (some of them are still on the market) was a step in the right direction, but the problems caused by non-specific interactions, loss of activity, low yield, and long production time were still not fully solved. The first experiments with newly developed chromatographic units based on stocked membranes (MemSeps, Millipore, Bedford, MA, USA) yielded promising separations, but some problems in terms of their construction and sample distribution at the inlet were only partially solved. However, the use of these kinds of separation units paved the way for the development of compact monoliths [40,41].

## 2. From Membranes to CIM Monoliths

### 2.1. Early Developments

At the end of the 1980s, Hjertén’s group developed a kind of monolithic stationary phase by compressing non-porous agarose beads that were successfully used for the chromatographic separation of proteins [40]. However, this kind of monolith was never commercially used [42]. Interestingly, work on the high-performance chromatography of proteins with compressed, non-porous agarose beads had already highlighted the feasibility of flow-rate-independent resolution in high-performance hydrophobic interaction chromatography (HIC) [40]. However, agarose-based monoliths never entered the market.

**Figure 1:**
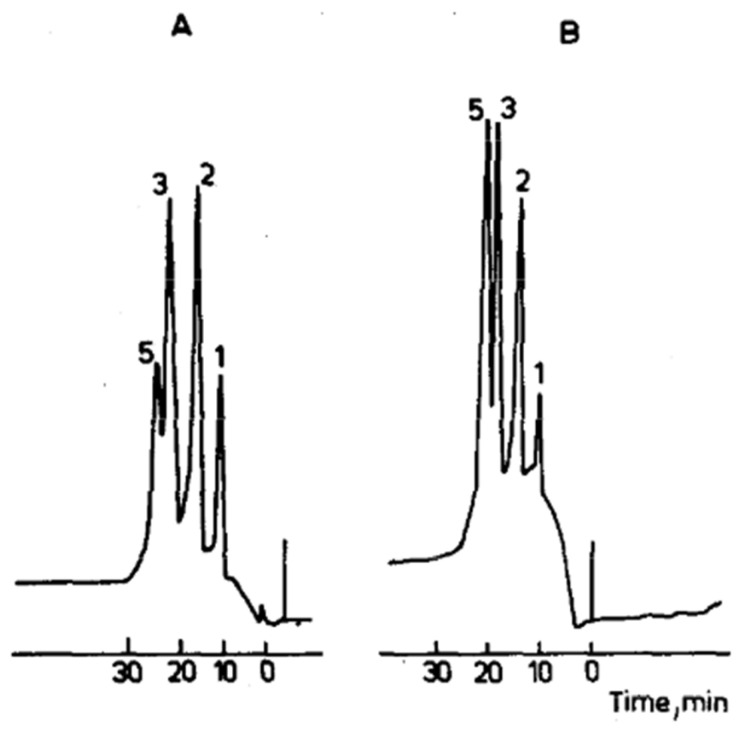
Protein separation using a G-50X8 membrane (1 mm thick, 20 mm diameter) at different loadings: (**A**) 5 mg and (**B**) 1.2 mg of total protein. Mixture components: (1) myoglobin, (2) ovalbumin, (3) lysozyme, and (5) chymotrypsinogen. Reprinted with permission from Ref. [43]. Copyright 1991 Elsevier. Only four proteins were in the mixture; the “5” over the chymotrypsinogen peak is likely an annotation error in the original paper.

The concept of compact porous membranes proposed by Tennikova et al. was firstly almost totally neglected [41]. In their pioneering work, the Russian–Czech group introduced macroporous polymer layers that were originally referred to as membranes, using them as a chromatographic unit for the separation of standard proteins (cf. Figure 1). It was also the reason that the first chromatographic separation demonstrated the possibility of using this kind of medium for separation, but it was still inferior in comparison to “classical” columns on the market. This chromatographic support had an enormous advantage: the epoxy-activated surface enabled additional in situ modification and separation in different chromatographic modes like ion exchange, RPC as well as the immobilization of different ligands for affinity chromatography. However, some important problems like the construction of the corresponding chromatographic hardware were not satisfactorily solved. In summary, the work by Tennikova et al. represented an essential step in the development of porous polymer membranes, essentially paving the way for the creation of monolithic stationary phases [41]. Their studies demonstrated the utility of polymer-synthesized membranes for protein separation but did not extend the work to other biological macromolecules. They used macroporous polymeric membranes, approximately 1 mm thick, that were synthesized from poly(glycidyl methacrylate-co-ethylene dimethacrylate), and the free epoxide groups were further derivatized to add different functional groups. Again, the separation of standard proteins was carried out on membrane units modified with sulfo- and hydrophobic C_4_- or C_8_- groups. Compared with HPLC separations using chromatographic columns in cation exchange and hydrophobic (C_4_ and C_8_) mode, the results were similar. The advantage of high-performance membrane chromatography (HPMC) is that the pressure used is lower by as much as two orders of magnitude than that of conventional HPLC columns. Consequently, very fast separations (up to two orders of magnitude faster) and high loading capacity can be reached. This makes HPMC suitable for both analytical and preparative separations [41].

### 2.2. First Experiments with Enzyme Immobilization on Monolithic Supports

**Figure 2:**
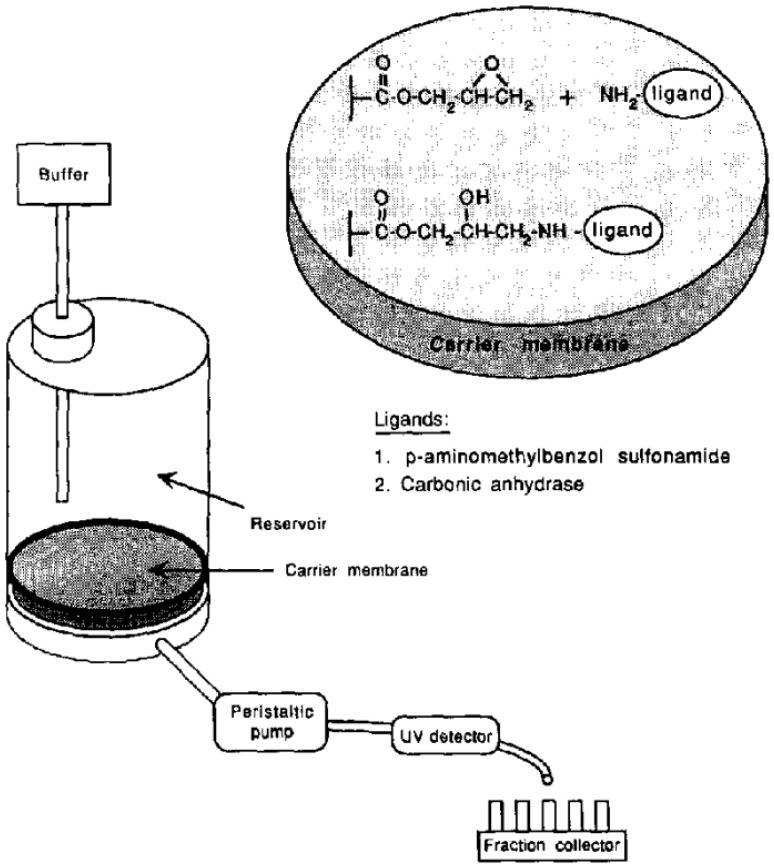
Structure and setup of ligand-functionalized membranes for flow-through analysis. Reprinted with permission from Ref. [44]. Copyright 1991 Elsevier.

**Figure 3:**
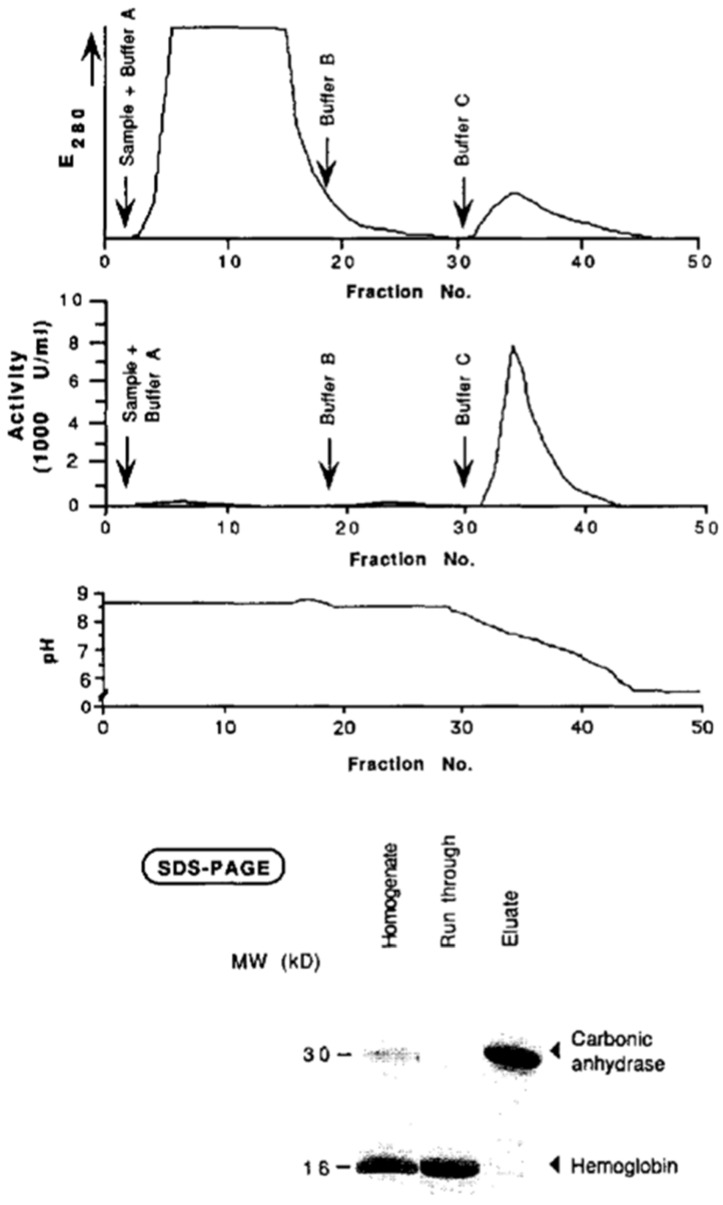
Purification of carbonic anhydrase from human erythrocyte lysate via affinity chromatography. After equilibration and washing, elution was performed at pH 5.5 with 1 M NaCl. Protein fractions were verified by SDS-PAGE. Reprinted with permission from Ref. [44]. Copyright 1991 Elsevier.

**Figure 4:**
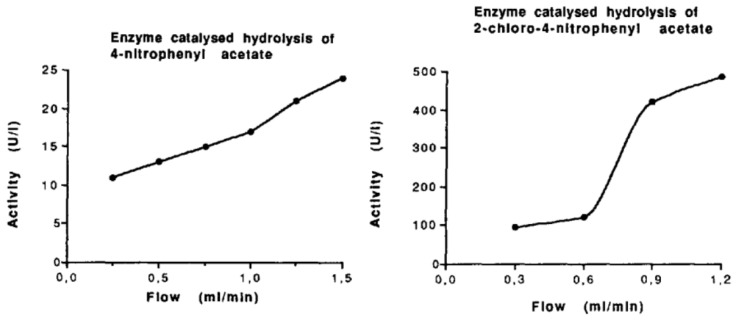
The influence of flow rate on enzymatic activity was studied using carbonic anhydrase immobilized on a monolithic membrane. Two substrate solutions were pumped through the membrane at varying flow rates, and an increase in enzymatic activity was observed for both substrates at higher flow rates. Reprinted with permission from Ref. [44]. Copyright 1991 Elsevier.

Our first experiments with the immobilization of (low molecular weight) ligands on epoxy-activated monolithic membranes and their use for the purification of active carbonic anhydrase are demonstrated in Figure 2. The statement in this work that the “use of membrane supports as stationary phase, coupled with ligands of choice, allows all kinds of chromatography and offers a powerful alternative to both soft gel chromatography and high-performance liquid chromatography” is still valid (see above). The enzyme carbonic anhydrase was isolated from hemolysates of human erythrocytes using affinity chromatography on a monolithic unit (this time still called a “membrane”) (cf. the corresponding Figure 3). The isolated enzyme was immobilized on an epoxy-activated chromatographic unit (again called “membrane”), and kinetic investigations were carried out using 4-nitrophenyl acetate and 2-chloro-4-nitrophenyl acetate as the substrates (cf. next Figure). These basic experiments with two different substrates demonstrated that enzymatic conversion was not limited by the flow rate and that the convection, and not diffusion, was responsible for the rate of enzymatic conversion (Figure 4). However, the “chromatographic unit” shown in Figure 2 is insufficient for routine use neither for chromatographic separations nor for “in-flow” enzymatic reactors.

Only several years after we introduced the concept of monolithic disk enzyme reactors [44], in 1996, Petro et al. constructed the first monolithic column enzyme reactor using a polymerized continuous rod as the support, which showed improved performance over traditional packed-bead reactors [45]. These combined works highlight how convective flow through a porous monolith can overcome the diffusion limitations that faced particulate supports, setting the stage for the widespread interest in monolithic reactors for enzymatic conversion.

Monolith reactor designs have evolved significantly since their inception, with advancements in format and configuration aimed at improving flow and usability. Early designs were relatively simple disks or single-column monoliths, and, in the mid-2000s, capillary-based monolithic enzyme microreactors were developed to enable high-throughput microscale reactions and direct coupling to analytical instruments [46]. Innovations in in situ polymerization allowed enzymes to be immobilized during monolith formation in the capillaries, simplifying reactor preparation to a one-step process [46]. Recent years have seen monolith designs tailored for specific applications, for example, 3D printing technology has been applied to fabricate custom-shaped monolithic microreactors that withstand high pressures and integrate seamlessly into flow systems [47,48]. These design improvements, ranging from format miniaturization to novel fabrication methods, have increased the versatility and integration of monolithic enzyme reactors into various processing setups. The “rod-like” monolithic column is still use, but only on an analytical scale. It is an excellent solution and enables miniaturization. However, for preparative separation on an industrial scale, a different solution had to be found.

Over time, the materials used for monolithic enzyme supports have diversified, leading to better chemical compatibility and enzyme attachment strategies. Early monoliths were predominantly organic polymer matrices (e.g., polyacrylamide or glycidyl methacrylate-co-ethylene dimethacrylate), valued for their ease of functionalization with groups that could covalently bind enzymes [49]. In the 2000s, inorganic silica monoliths emerged as alternatives, offering high mechanical rigidity and well-defined porous structures. These silica-based monoliths could be surface-modified (e.g., with epoxides or amines) to immobilize enzymes and had similarly efficient flow-through characteristics [50]. Organic–inorganic hybrid monoliths were also developed, combining polymer networks with silica or other inorganic components, to harness the functional group versatility of polymers and the mechanical strength of inorganic supports [51]. Moreover, surface chemistry techniques have been refined to improve the enzyme loading on and stability of monoliths, significantly broadening the types of enzymes that can be immobilized on these supports [52]. This progression in materials science has led to the production of monolithic reactors that are chemically robust, easily derivatized for enzyme attachment, and compatible with a wide range of reaction conditions.

A key advantage driving the adoption of monolith enzyme reactors is their higher catalytic efficiency relative to that of traditional immobilized enzyme systems. Like in the process of chromatographic separation, the monolith’s interconnected macroporous structure permits the convective flow of substrates through the support, effectively eliminating the diffusional mass transfer limitations and allowing enzymes to operate at high turnover rates even at high flow speeds [44,45,49,53]. Enzymes immobilized on monoliths often retain higher activity and can be used for rapid reactions, for instance, proteolytic digestion that traditionally took hours in solution could be completed in minutes using a monolithic microreactor, achieving orders of magnitude speed-ups [47]. Additionally, immobilization on monolith surfaces tends to improve enzyme stability and enables the reuse of the biocatalyst over many reaction cycles, increasing the overall efficiency of the process [54]. Thus, optimized pore structures and immobilization chemistries can ensure high productivity, low backpressure, and the stable operation of monolithic reactors.

Throughout the years, monolithic enzyme reactor technology has expanded into the pharmaceutical and biotechnology sectors, where immobilized enzyme monoliths are used, among others, for drug metabolite screening, inhibitor discovery, and various biotransformation processes [50]. Monolithic enzyme systems have also been integrated into biosensors and microanalytical devices thanks to their small form factor and fast response, allowing the detection (and degradation) of substrates or toxins via enzyme-catalyzed reactions in flow [52,55]. Over the years, monolith reactors have evolved beyond laboratory-scale digestion with efforts focused on continuous processing and in-line biocatalysis, though scale-up challenges persist [49,56].

**Figure 5:**
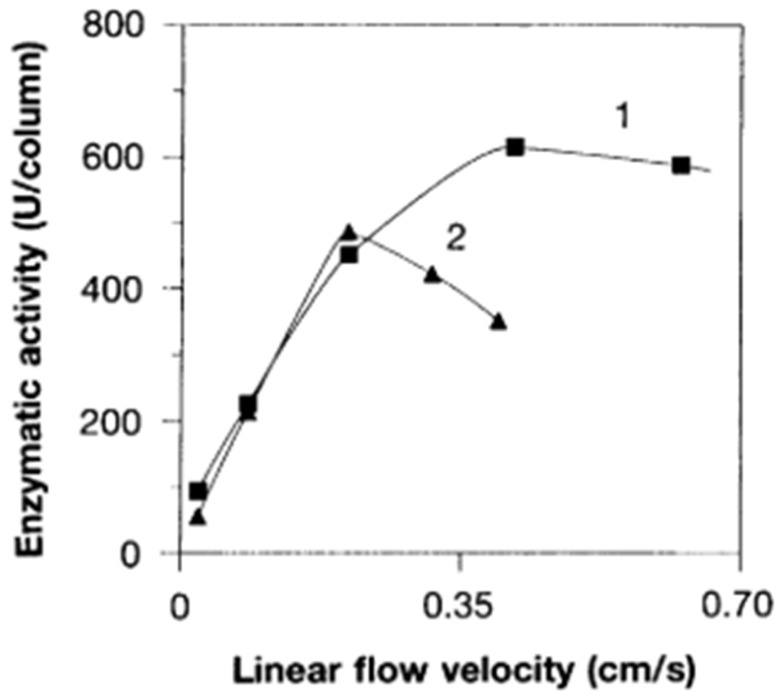
Effect of flow velocity on enzymatic activity of trypsin immobilized on rod (curve 1) and bead (curve 2) bioreactors. Reprinted with permission from Ref. [53]. Copyright 1996 American Association for the Advancement of Science.

The rod-like monolithic column introduced in Ref. [53] is still used but only on an analytical scale. It is an excellent solution and enables miniaturization. However, they have only limited use for preparative separation at the industrial scale. As shown in Figure 5 (below), they have advantages over bead reactors, but the rod like geometry still limits their use at higher flow rates (cf. Figure 5). In order to solve this problem, different and new methods had to be found.

### 2.3. The Way Toward Convective Interaction Media

**Figure 6:**
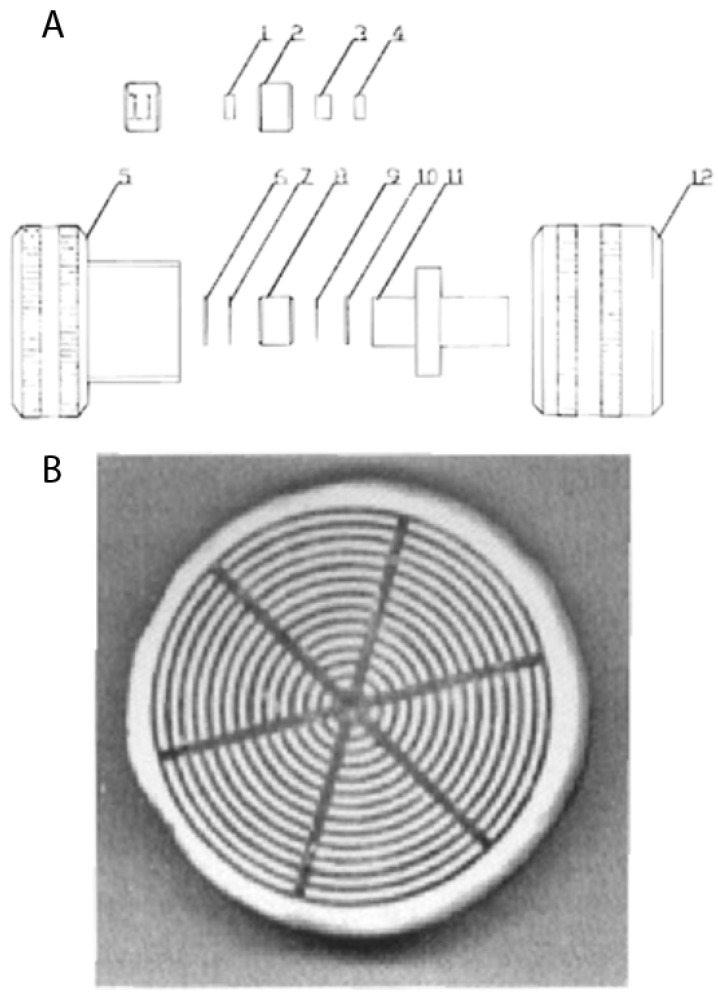
(**A**) Schematic diagram of a compact porous disk assembly (10 mm diameter, 3 mm thick), showing holder elements (labeled 1–12). (**B**) Visualization of sample distribution with ferritin immobilization on an epoxy-activated disk. Reprinted with permission from Ref. [57]. Copyright 1992 Elsevier.

The problem of sample distribution by use of disk-shaped membranes was recognized in our early work [57]. In order to document sample distribution using different forms of newly constructed distribution units, a ferritin solution was injected into an epoxy-activated disk, and the optimal form of the newly constructed one was chosen (see Figure 6). It also enabled the controlled chromatographic separation of standard proteins and biological samples like human plasma and solubilized plasma membranes. However, the very fast chromatography of proteins and molecular transport (and later microparticle transport) by convection rather than diffusion was recognized and analyzed a few years later [58,59].

### 2.4. Scaling-Up and Construction of Radial Flow Monolithic Columns

**Figure 7:**
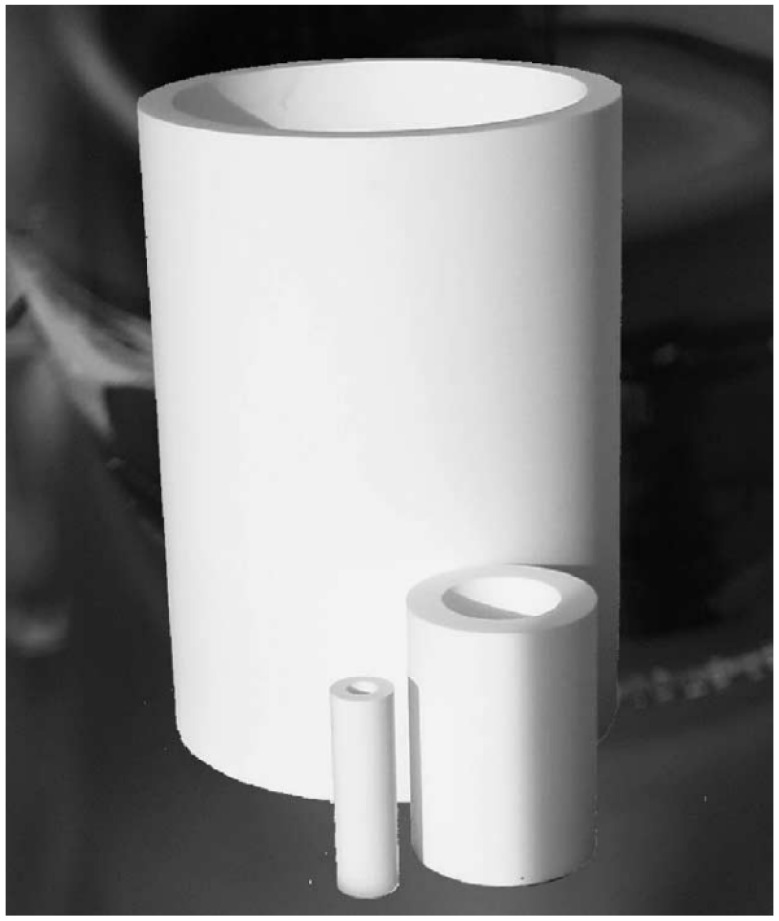
Radial flow CIM monolithic columns of different volumes (80, 800, and 8000 mL prototype), illustrating scalable designs for industrial applications. Reprinted with permission from Ref. [17]. Copyright 2005 Elsevier.

The next logical step was to scale-up monolithic columns to the industrial scale, which was successfully accomplished. In order to keep a large surface for sample and buffer entrance and distribution, the radial chromatography mode was chosen [60], and the term convective interaction media (CIM) for both disk- and cylinder-shaped monoliths was created. Modern examples of such large-scale monoliths are shown in Figure 7 [17].

**Figure 8:**
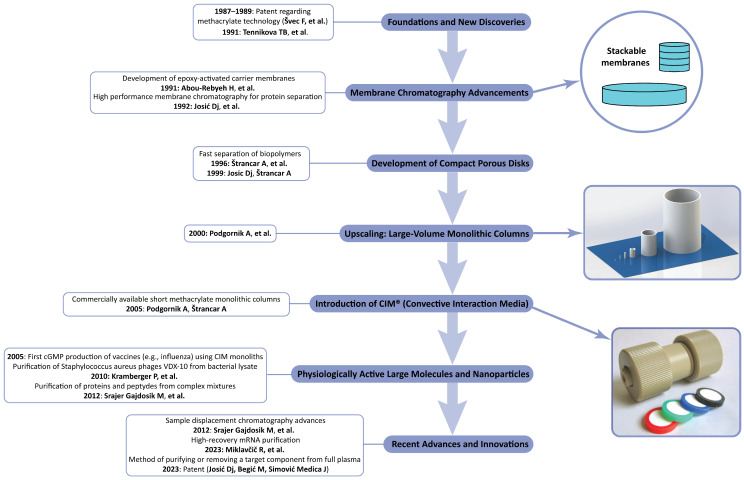
Key milestones in the historical development of monolithic (later CIM monolithic) chromatography (Refs. [6,43,44,57,58,59,60,67,68,69,70,71]).

A review published by František Švec [61] gives a general overview about the use of monolithic supports at the analytical and preparative scales. The present review is focused on the use of polymethacrylate-based CIM monoliths for the separation of large biopolymers and nanoparticles and the in-process control of industrial-scale productions. We already demonstrated that compact, polymer-based monolithic supports can be used for both the large- and small-scale separations of biologically active macromolecules as well as nanoparticles and viruses. Their high efficiency and selectivity, together with their relatively small surface, makes them almost ideal for the newly developed chromatographic techniques like sample displacement chromatography, which was also presented in our last short overview dedicated to monoliths [62]. Ma et al. [63] gave a complete overview of the modern use of CIM monolith-based enzyme reactors. This period was also characterized by the use of monoliths for the separation and isolation of macromolecules like large proteins and nucleic acids. It was also the starting period for the increased use of monoliths for the separation of microparticles, especially plasmids, extracellular vesicles, and whole viruses [64,65,66]. These developments are shown in Figure 8.

**Figure 9:**
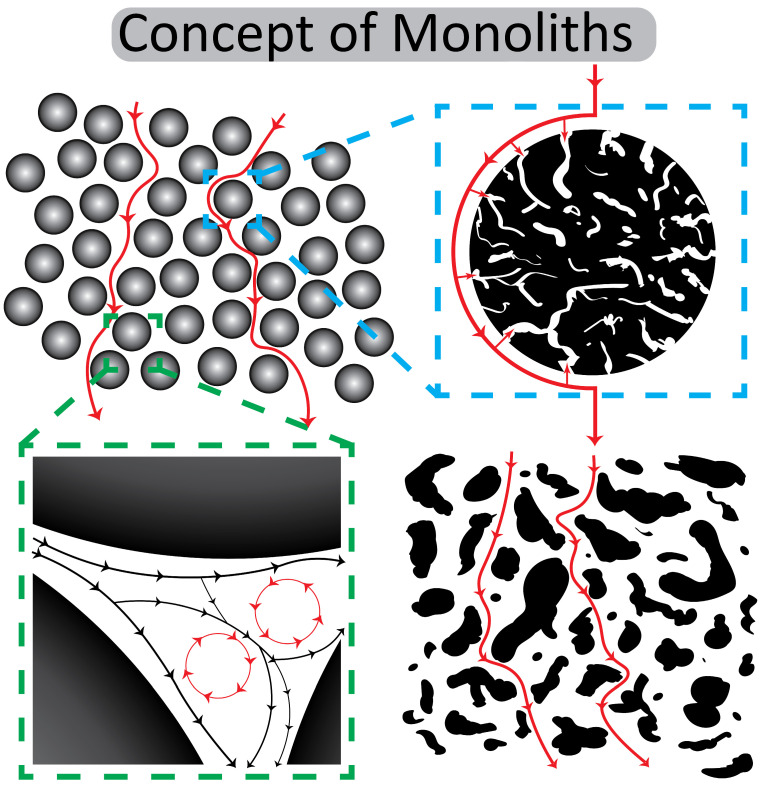
A conceptual comparison of bead-based and monolithic chromatography. The top left panel illustrates typical bead-based chromatography with spherical particles and flow paths around them. The top right panel shows a magnified cross-section of a single porous bead. The bottom left panel depicts the spaces between the beads and the formation of eddy currents in a packed bed. In contrast, the bottom right panel highlights the interconnected, continuous structure of a monolith and the convective flow paths within its pores. Black and red lines represent the principal flow paths illustrated in the schematic.

The reason for this development is the practically absent diffusion in this kind of monolithic structure and the material exchange by convective flow in their channels (cf. Figure 9) but also the enormous progress in the surface optimization and exact modelling of channel diameters. It enables direct interactions between the ligands that are immobilized on the channel surface of monoliths and the ligates in the mobile phase, practically independent of the diffusions constants of the ligate (cf. Table 1 and Ref. [65]). Even large viruses like hepatitis B virus (HBV) can penetrate the large-diameter channels of CIM monoliths and bind to the ligate on the surface [72].

### 2.5. Applications

In an earlier study, monolithic supports were implemented at the production scale for high-performance plasmid DNA purification under cGMP conditions, thus overcoming diffusion limitations, allowing fast flow rates, and ensuring consistent product quality [17]. Soon thereafter, it was shown that the use of short monolithic columns substantially reduced virus purification times from days to mere hours while achieving high recovery rates, highlighting the use of such supports in large-virus processing [73]. Extending this principle, work on the separation of multiple plant viruses from mixed samples demonstrated that sufficiently differing charge properties permit efficient resolution on monoliths, thus enabling selective downstream separation (and purification), even for closely related virus strains [74]. Recent studies confirmed the versatility of monolithic media for recombinant oncolytic NDV, SARS-CoV-2, and vesicular stomatitis virus, while still achieving high recovery at scale [75,76,77]. In another study, an automated online system integrated immunoaffinity-based monolithic columns with asymmetrical flow field–flow fractionation for the rapid isolation and fractionation of complex plasma-derived nanosized biomacromolecules [78]. Specifically, anti-apolipoprotein B-100 and anti-CD9 or anti-CD61 antibodies on monolithic disks enabled the enrichment of lipoproteins and extracellular vesicles (EVs), followed by asymmetrical flow field–flow fractionation (AsFlFFF) of EV subpopulations. The need for more efficient separations is particularly relevant given that lipoproteins and EVs can overlap in size and density, making their separation generally challenging [79]. Notably, EV isolation by a monolithic immunoaffinity approach immobilized with anti-CD61 antibody showed that platelet-derived EVs could be purified in under 20 min, with minimal contamination, and the column was reusable for multiple runs [80]. More recently, spongy-like monoliths hybridized with TiO_2_ offered exceptional speed and comprehensiveness for EV enrichment, involving a single-step purification and achieving superior yield [16].

Monolithic supports have significantly advanced the purification of plasmid DNA, viruses, and EVs, which is crucial for gene therapy and vaccine development, and is now being adopted for emerging mRNA therapeutics [81,82]. Multiple studies have demonstrated that monoliths with a large channel diameter overcome diffusion constraints, enabling the fast, high-resolution separation of plasmid isoforms [83,84,85,86]. The same principle drastically reduced virus purification times from days to mere hours while preserving infectivity [73]. Recent work using the surface-initiated ATRP grafting of linear polymethacrylate chains increased the dynamic binding capacity for a 7.3 kbp plasmid from 1.8 mg mL^−1^ on non-grafted CIM DEAE to 15 mg mL^−1^ on the grafted column while still achieving ≥95% elution recovery and complete RNA clearance [87]. Closely related plant viruses were likewise resolved using subtle charge differences, showcasing monoliths’ capacity for precise virus separations [74]. Further extending these applications, immunoaffinity-based monolithic disks have been integrated with asymmetrical flow field–flow fractionation for the rapid isolation of EVs from complex plasma matrices [78]. Another interesting approach employing anti-CD61-functionalized monoliths enabled EV purification in under 20 min and permitted multiple column reuses [80]. More recently, spongy-like hybrid monoliths have expanded these capabilities by offering rapid, single-step EV purification with superior yield [16]. Modern monolith developments also directly support viral- and plasmid-based gene therapy, as well as RNA vaccine platforms, all of which largely benefit from mild yet efficient downstream processes [84,88,89]. By operating at low backpressure, monoliths allow large-scale manufacturing and help maintain the structural integrity of sensitive targets such as enveloped viruses and mRNA constructs [60,90]. Together, all these advancements align well with the stringent needs of modern biopharmaceuticals, allowing high throughput without compromising product quality.

### 2.6. In-Process Analyses

In-process analytics have significantly evolved through high-throughput strategies coupling monolith-based separations with automated robotic platforms and standard 96-well microtiter plates [62]. Affinity-based monolithic supports integrated into robotic pipetting stations enabled the rapid, simultaneous isolation of various immunoglobulins from human serum [62,91]. Similar platforms were successfully applied for the high-throughput isolation and glycosylation analysis of IgG from thousands of plasma samples using monolithic 96-well plates with immobilized protein G [92]. Comparable semi-automated workflows were reported for transferrin and fibrinogen, demonstrating the broader applicability of monolithic immunoaffinity platforms [93,94].

### 2.7. Direct Application of Undiluted Biological Fluids and Cell Culture Supernatants

The first step in the chromatographic purification of biological fluids, like body fluids or cell culture supernatants, is the loading of the sample on the chromatographic unit. Frequently, ion-exchange-based chromatographic supports are used for the capture step. However, the salt concentrations in samples like biological fluids and cell culture supernatants before loading to the separation unit are too high. In order to lower the salt concentration, and consequently the optimal binding of the target substance(s), an extensive dilution or ultrafiltration/diafiltration step is necessary [95]. Consequently, the use of chromatographic supports that can bind target components without additional processing steps like ultrafiltration and dilution is needed. Wide-channel CIM monoliths with ion-exchange or mixed-mode ligands are ideal supports for this type of chromatography process. It was demonstrated that cryopoor and full human plasma could be loaded and processed on an anion exchange CIM monolithic column without dilution. It was the first step in the development of a continuous process for plasma fractionation and the isolation of therapeutic proteins [67,96].

**Figure 10:**
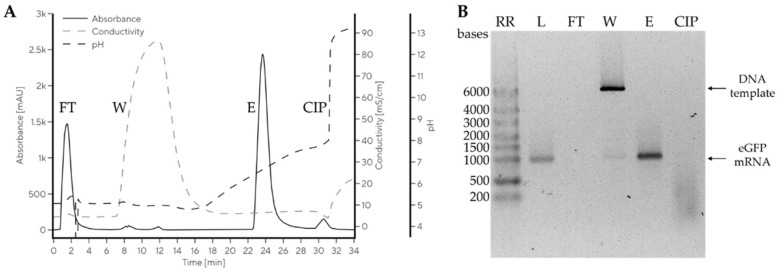
Purification of mRNA from the IVT reaction mixture using a CIMmultus Swiper (Sartorius BIA Separations, Ajdovščina, Slovenia) 1 mL column. Preparative chromatogram (**A**) and agarose gel electrophoresis (AGE) analysis of the collected fractions (**B**). Reprinted from Ref. [68]. 2023 MDPI (CC BY 4.0).

Miklavčič et al. used multimodal CIM monolithic chromatography media for the isolation of mRNA at neutral pH and room temperature [68]. A baseline separation of large mRNA (up to 10,000 nucleotides from the parental plasmid DNA) was achieved (cf. also Figure 10). This developed CIM monolith enabled the mass transfer of these large molecules with a yield of 80% while preserving their integrity and ensuring high stability after the isolation process.

Additional previous and current key references for various types of chromatography on monolithic supports are given in Table 2.

## 3. Outlook and Vision for the Future

Regarding the direct application of undiluted samples to a monolithic chromatographic unit with minimal manipulation, the first steps were already achieved with newly developed fractionation processes for human plasma [67,95]. These experiments paved the way for the direct in situ removal of pathological molecules from body fluids [96].

Sample displacement was achieved in a developed process for the isolation of clotting factor IX from cryopoor human plasma [67] and mRNA [68]. It was also demonstrated that this strategy could be used for the isolation of other therapeutic proteins and other physiologically active macromolecules from complete, undiluted human plasma [95] and cell culture supernatants [68].

Regarding new solutions in continuous chromatography, we already demonstrated that both disk- and cylinder-shaped monolithic supports can be used in so-called conjoint sequence for the capture of diverse target macromolecules. Furthermore, our experience with the application of annular chromatography and the use of newly developed strategies for this process shall be used for further development [96,97].

Tailored chromatographic materials with both optimized channel surfaces and diameters are already used in recently developed processes for the chromatography of undiluted human plasma [67,95] and for the isolation of human mRNA (see Figure 10 and Ref. [68]).

## Figures and Tables

**Table 1:** The diffusion constants (*K*_diff_) of selected biomolecules and the approximate diameters of the selected viral particles, illustrating the relationship between the molecular size, diffusivity, and suitability of convective monolithic supports for large biomolecule chromatography. Adapted from the work of P. Gagnon (2008) (Ref. [65]).

Diffusion Constants for Selected Solutes			Approximate Diameters of Selected Viral Particles	
Solute	Size	*K*_diff_	Virus	Diameter
Light chain	23 kDa	9.1 × 10^−7^	AAV	20–26 nm
BSA	66 kDa	6.7 × 10^−7^	MVM	25 nm
IgG	150 kDa	4.9 × 10^−7^	Rhinovirus	30 nm
Urease	480 kDa	3.5 × 10^−7^	HBV	42 nm
IgM	960 kDa	2.6 × 10^−7^	Adenovirus	59–67 nm
ETX	2 MDa	2.1 × 10^−7^	EBV	80–100 nm
CMV	5 MDa	1.2 × 10^−7^	HIV	100–120 nm
TMV	40 MDa	5.0 × 10^−8^	HSV	110–200 nm
DNA_1_	4.4 kbp	1.9 × 10^−8^	MuLV	120–150 nm
DNA_2_	33.0 kbp	4.0 × 10^−9^		

ETX = endotoxin; CMV = cucumber mosaic virus; TMV = tobacco mosaic virus; AAV = adeno-associated virus; MVM = minute virus of mice; HBV = hepatitis B virus; EBV = Epstein–Barr virus; HIV = human immunodeficiency virus; HSV = herpes simplex virus; MuLV = murine leukemia virus.

**Table 2:** Representative key references for various types of chromatography on monoliths.

Chromatography Type	Selected References
High-performance “membrane” chromatography	Tennikova, T.B.; Svec, F.; Belenkii, B.g. High-Performance Membrane Chromatography. A Novel Method of Protein Separation [41]. *Journal of Liquid Chromatography* **1990**, *13*, 63–70 Josić, D.; Reusch, J.; Löster, K.; Baum, O.; Reutter, W. High-Performance Membrane Chromatography of Serum and Plasma Membrane Proteins [57]. *Journal of Chromatography A* **1992**, *590*, 59–76
Affinity chromatography	Abou-Rebyeh, H.; Körber, F.; Schubert-Rehberg, K.; Reusch, J.; Josić, Dj. Carrier Membrane as a Stationary Phase for Affinity Chromatography and Kinetic Studies of Membrane-Bound Enzymes [44]. *Journal of Chromatography B: Biomedical Sciences and Applications* **1991**, *566*, 341–350 Neumair, J.; D’Ercole, C.; De March, M.; Elsner, M.; Seidel, M.; de Marco, A. Macroporous Epoxy-Based Monoliths Functionalized with Anti-CD63 Nanobodies for Effective Isolation of Extracellular Vesicles in Urine [64]. *IJMS* **2023**, *24*, 6131.
Ion exchange chromatography	Podgornik, A.; Barut, M.; Štrancar, A.; Josić, D.; Koloini, T. Construction of Large-Volume Monolithic Columns [60]. *Anal. Chem.* **2000**, *72*, 5693–5699 Kramberger, P.; Peterka, M.; Boben, J.; Ravnikar, M.; Štrancar, A. Short Monolithic Columns—A Breakthrough in Purification and Fast Quantification of Tomato Mosaic Virus [73]. *Journal of Chromatography A* **2007**, *1144*, 143–149
Hydrophobic interaction chromatography	Svec, F.; Fréchet, J.M.J. New Designs of Macroporous Polymers and Supports: From Separation to Biocatalysis [53]. *Science* **1996**, *273*, 205–211 Sviben, D.; Forcic, D.; Ivancic-Jelecki, J.; Halassy, B.; Brgles, M. Recovery of Infective Virus Particles in Ion-Exchange and Hydrophobic Interaction Monolith Chromatography Is Influenced by Particle Charge and Total-to-Infective Particle Ratio [66]. *Journal of Chromatography B* **2017**, *1054*, 10–19
Sample displacement chromatography	Srajer Gajdosik, M.; Clifton, J.; Josic, D. Sample Displacement Chromatography as a Method for Purification of Proteins and Peptides from Complex Mixtures [6]. *Journal of Chromatography A* **2012**, *1239*, 1–9 Černigoj, U.; Martinuč, U.; Cardoso, S.; Sekirnik, R.; Krajnc, N.L.; Štrancar, A. Sample Displacement Chromatography of Plasmid DNA Isoforms [83]. *Journal of Chromatography A* **2015**, *1414*, 103–109

## Data Availability

Data are contained within the article.
